# Comparison of the Effects of TheraCal LC and Mineral Trioxide Aggregate on Direct Pulp Capping (DPC) Based on Histological Findings

**DOI:** 10.7759/cureus.25326

**Published:** 2022-05-25

**Authors:** Basel Zeater, Souad Abboud, Louay Hanafi

**Affiliations:** 1 Endodontic Dentistry, Damascus University, Damascus, SYR; 2 Pediatric Dentistry, Damascus University, Damascus, SYR

**Keywords:** theracal lc, resin tag calcium silicate, mineral trioxide aggregates, premolars, direct pulp capping

## Abstract

Background: Direct pulp capping can conserve its vitality by placing materials that promote dentin bridge (DB) formation at the exposure site. This study aimed to determine whether TheraCal LC could produce a layer of reparative dentin. It also compared the histological differences between treatment with mineral trioxide aggregate (MTA) and TheraCal LC.

Material and methods: A sample of 20 maxillary and mandibular premolars, which had previously been indicated for extraction in orthodontic therapy, was taken from 10 patients and randomly divided into two halves, a TheraCal LC, and an MTA group. Pulpal exposure was achieved by similar class I preparations, which were restored with a resin-modified glass-ionomer and extracted after ten weeks, noting that these interventions have been performed on live teeth in the oral cavities. The newly formed dentin bridge thickness, the inflammation degree within the pulp tissue, and odontoblast function were thoroughly examined histologically and compared between the two groups using the Mann-Whitney test and an analysis software SPSS (statistical package for the social sciences, v.26, IBM, New York, N, USA), at a significance level of a=0.05.

Results: Dentin bridge composition in the TheraCal group had 80% effective tubules and 20% defective tubules, while in the MTA group, the proportions were 90% and 10%, respectively. Dentin bridge thickness in the TheraCal group was greater than 0.25 mm in 60%, and 0.1-0.25 mm in 40% of the sample compared to the MTA group, which had 70% greater than 0.25 mm, and 30% between 0.1 and 0.25 mm in dentin bridge thickness. Statistically, there was no significant difference between both groups (P=0.739).

Conclusion: Statistically insignificant differences in dentin bridge composition and thickness produced by both TheraCal and MTA materials render them similar in their effectiveness in treating pulp exposures through pulp capping.

## Introduction

In restorative dentistry, maintaining pulp vitality is a significant objective. The goal of vital pulp therapy is to preserve the dental pulp viability while encouraging the residual pulp tissue to renew the dental-pulp complex [1]. Direct pulp capping (DPC) is a procedure that is used to promote pulp healing and dentin repair, and since choosing the suitable material is critical to successful treatment outcomes, many physicians and academics have studied capping materials [2]. The goal of producing such a dental bridge was the catalyst in developing and making improved pulp capping materials and techniques. These techniques are capable of inducing cellular proliferation, achieving well-sealed dentin, and generating a viable and stable dentin bridge. Among the many materials studied regarding different DPC materials, mineral trioxide aggregate (MTA) and TheraCal LC seem to predominate. MTA is one of the most critical materials used in dentistry, and today, it is used in many clinical and laboratory applications based on many studies, it has been considered the ideal and reference material (Gold Standard) [3]. Moreover, it showed an increase in fracture resistance when applied to simulated immature teeth [4]. It is composed of vital active silicate cement, which has been shown to be effective as a DPC material in both humans and experimental animals [5].

It is a material that has had high clinical success because of its relatively small molecular volume, sealing ability, high alkaline rate upon solidifying, and the slow release of calcium ions. Studies using MTA as a DPC material have shown its efficacy in inducing cellular proliferation, releasing cytokines, and forming a hydroxyapatite-like dentin surface [5,6].

TheraCal LC (Bisco Inc, Schaumburg, IL, USA) has been developed as a result of numerous studies examining the mechanical properties of calcium hydroxide. It has been generated from a light-cured resin-modified calcium hydroxide fortified with calcium silicate. This material gives TheraCal LC the capability to be used in many versatile roles. These include TheraCal LC to be used in both direct and indirect pulp capping and used as a liner or base under amalgam or composite restorations [7,8].

Many pulp capping materials such as calcium hydroxide, zinc oxide, and eugenol, bonding materials, resin modified glass ionomer, and MTA, have been used to secure a good seal for the dental pulp and isolate it from the oral cavity [9]. Calcium hydroxide has been the material of choice for pulp capping for decades and is considered the gold standard with its antibacterial properties. Still, some of the drawbacks of this material are its self-hardening, its ability to degeneracy, and lack of sealing [7], as dissolution of the capping material will create a vacuum between the restored material and the dental tissue, leading to tooth sensitivity following restoration, microscopic fossil leakage and subsequent failure of restoration [10].

Many studies have focused on the development of mechanical properties of calcium hydroxide, as there have been insufficient studies to evaluate the efficacy of theracal LC in direct pulp capping [8]. The purpose of this study is to determine if treatment with TheraCal LC results in a layer of reparative dentin and to examine whether any histological differences are present between treatment with MTA and TheraCal LC.

The null hypothesis is that there is no significant difference between MTA and TheraCal LC in dentine bridge composition, dentine bridge thickness, pulpal inflammation, or odontoblast layer.

## Materials and methods

Study design and sittings

This study is a randomized progressive double-blind prospective study composed of clinical and histological phases. It was approved by the ethics committee (number 3396) in the Faculty of Dentistry on May 1, 2015, and funded by Damascus University. This trail was conducted in accordance with the principles for medical research involving human subjects, as described by the Declaration of Helsinki.

Patients' recruitment and study flow

Ten patients from the Orthodontics Department at Damascus University, School of Dentistry, gave a written consent form before starting any treatment and after a thorough explanation of this research. The patients were all under 25 years of age.

Sample size calculation

The sample size was chosen based on previously published papers in the literature, where a sample of 20 maxillary and mandibular premolars was taken from 10 orthodontic patients, with two symmetrical teeth from each patient. All the teeth were caries-free, restoration-free, had well-closed apices, unbonded with brackets, and without orthodontic force applied by the orthodontist.

TheraCal LC was applied to one patient's teeth and MTA to the other. This was done randomly by the Excel program (Excel for Windows, v 2021, Microsoft Corporation, Washington, USA) without informing the patients and the dental histologist (examiner) which of the two substances had been used on each of their premolars.

Clinical work

**Figure 1:**
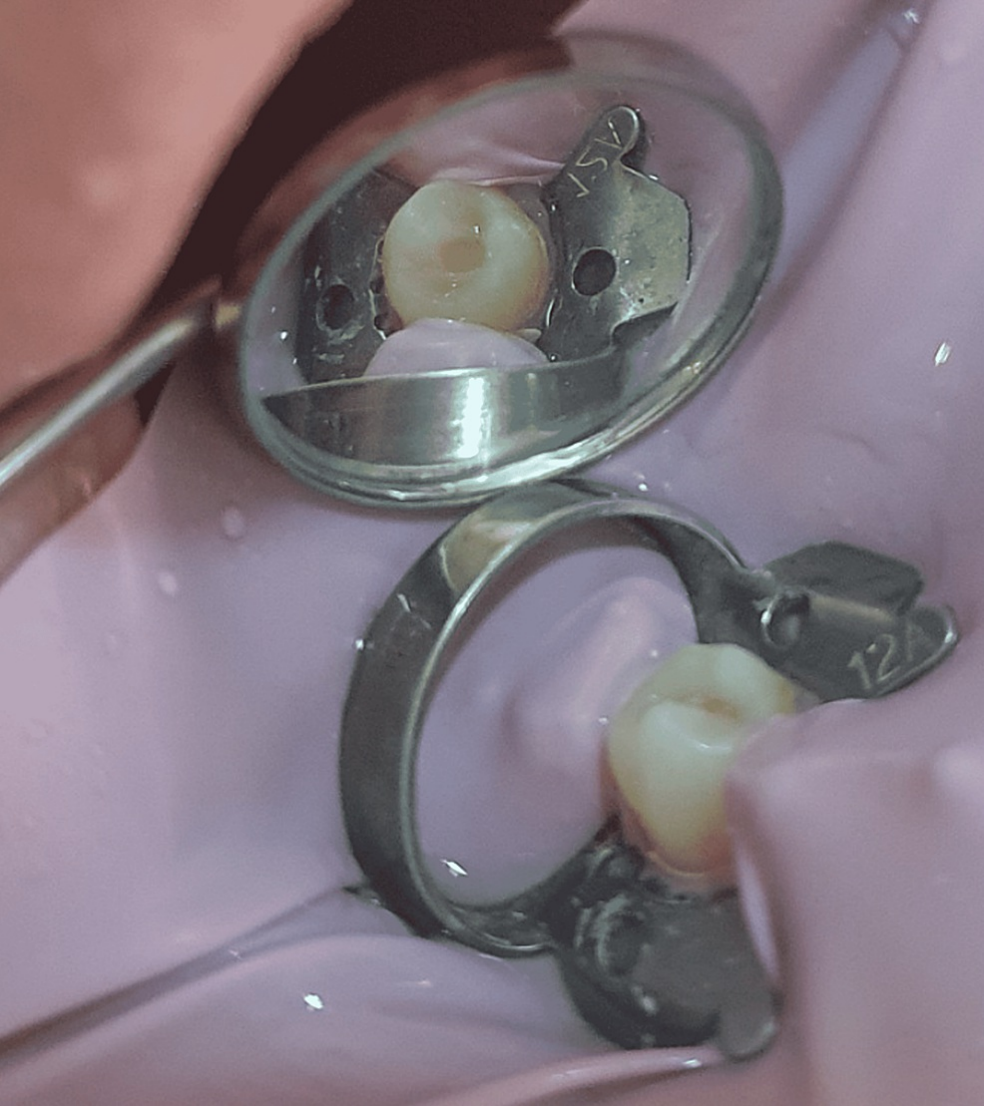
Class I preparation on a mandibular first premolar.

After administering anesthesia, a rubber dam was placed, and a Class I preparation was achieved on all teeth by using a diamond carbide bur (Figure 1).

Pulpal exposure was achieved by applying pressure from a stainless-steel explorer on the preparation floor. Sodium hypochlorite 2.5% was used to control bleeding, disinfect the site, and remove any remaining dentin [11]. Next, one of two studied materials (TheraCal LC, MTA) was applied on the teeth, which were already randomly selected, to simulate the DPC procedure.

TheraCal LC Group

The first group of 10 teeth was treated with TheraCal LC (Bisco Inc, Schaumburg, IL). The TheraCal liner was applied to the wet dentin. The layer was less than 1 mm in thick with a 2 mm diameter surrounding the exposure site. No contact with enamel was made. It was light-cured for 20 seconds, as indicated by the manufacturer.

MTA Group

For the second group, MTA (Dentsply, TULSA, OK, USA) was used. It was prepared using the manufacturer’s recommendation by mixing the powder with sterilized saline in a ratio of 3:1, followed by using wet cotton to achieve a strong cure.

These preparations were then restored with a resin-modified glass-ionomer (Vitrebond, 3M ESPE Dental Products, St. Paul, MN, USA). Three tests were administered to each of the 20 teeth to ensure all teeth had a healthy and vital pulp. These tests included a cold test (Teste de Vitalidade Endo Ice, Maquira, Germany), an ethyl chloride test, and a percussion test followed by extracting the samples, which completed 10 weeks after the initial glass ionomer restoration.

Histological work

**Figure 2:**
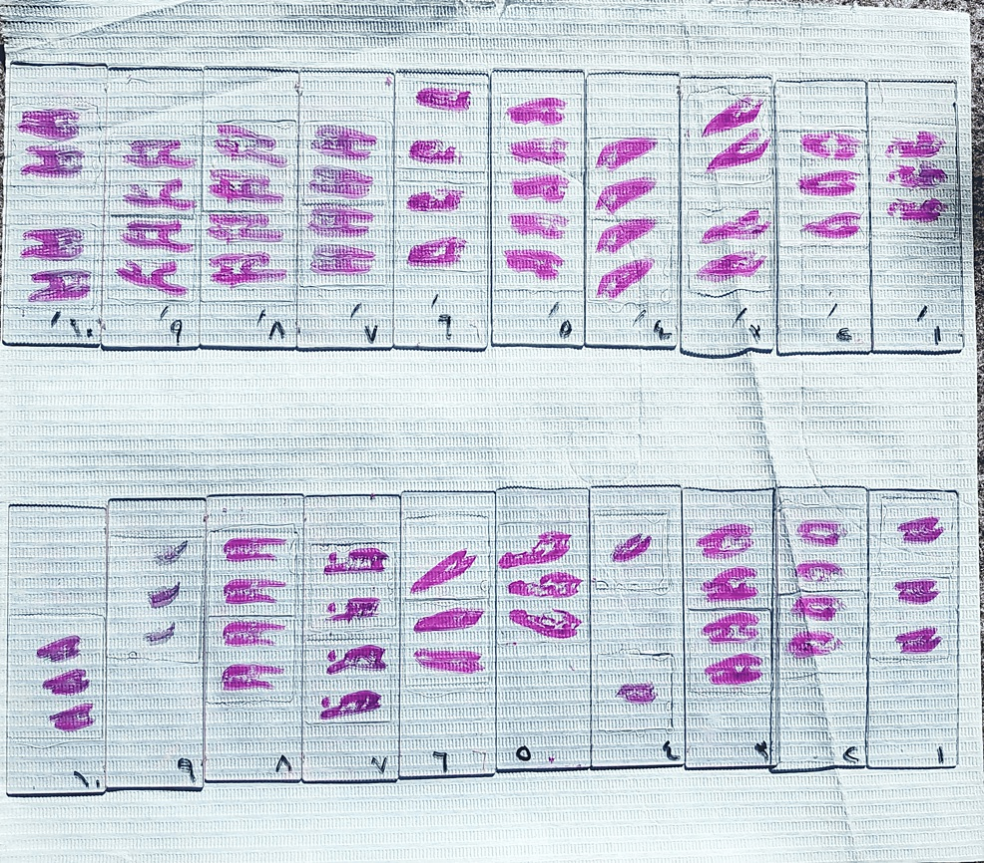
The histological sections of samples.

After the samples extraction done due to orthodontic treatment, they were immediately placed in a Formalin 10% suspension to preserve and fix the histological samples. Finally, the samples were sent to the pathology laboratory (Oral Pathology Department, Faculty of Dentistry, Damascus University) for preparing the sections for histological evaluation of the newly formed dentin bridge (Figure 2).

The histological study was carried out according to the following stages. Firstly, samples decalcification done by nitric acid solution 15% with formalin 10%, followed by washing them with running water. Secondly, drying all samples by alcohol with gradient concentration (50%, 70%, 95%) respectively. Then, several baths of Xylol were done to remove the excessive amount of alcohol. Thirdly, merging the samples into paraffin, then transferring them to pure and melted paraffin at a temperature of 55°C. Next, the paraffin molds were cut by using a microtomizer in order to obtain slices with a thickness of 4-6 microns. Last but not least, sections were moved to glass slides and then dried at 90°C for 10 minutes. Finally, samples were stained using the hematoxylin-eosin method and became ready to be studied under the microscope.

Outcome measures

A thorough examination of the newly formed dentin bridge was conducted on the extracted teeth by using an optical microscope (Olympus, Italy) with an attached digital camera. Four main items were studied; the composition of the dentin bridge, the thickness of the dentin bridge, the degree of pulpal inflammation, and the odontoblast layer.

Statistical analysis

The data were collected and recorded on the Excel program (Excel for Windows, v 2021, Microsoft Corporation, Washington, USA), while the statistics test was completed using SPSS V.26 (statistical package for the social sciences, v.26, IBM, New York, N, USA), and the significance level was set at a=0.05. A Mann-Whitney U test, which is used for group differences (two groups) with ordinal variables (the same variables used in this research), was conducted to study the difference between the composition and thickness of the newly formed dentin bridge, the degree of inflammation within the pulpal tissue, or the odontoblasts layer in the two groups.

## Results

Composition of the dentin bridge

**Figure 3:**
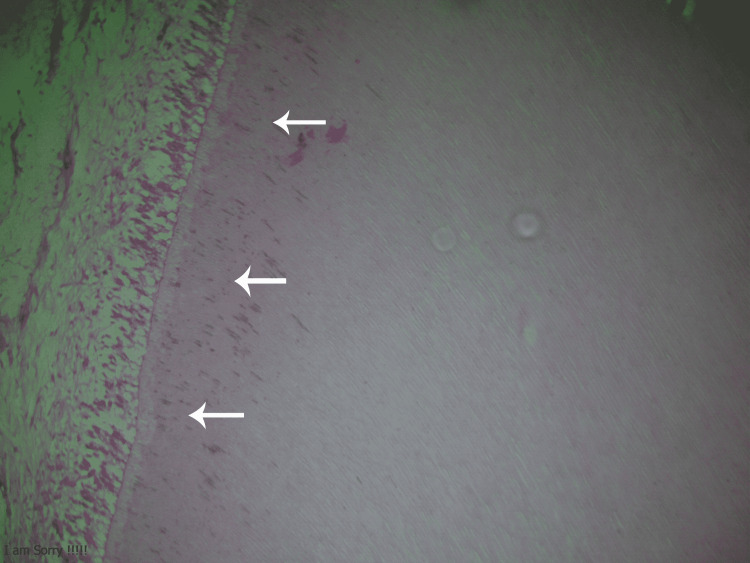
A histological preparation of a first premolar where TheraCal LC was applied showing the dentin bridge composition (H&E stain, 100×). H&E = hematoxylin and eosin.

The dentin bridge in the TheraCal group was composed of 80% effective tubules and 20% defective tubules. In comparison, in the MTA group, it was composed of 90% effective tubules and 10% defective tubules. This slight change in composition was statistically insignificant (P=0.739) (Figure 3).

Thickness of the dentin bridge

**Figure 4:**
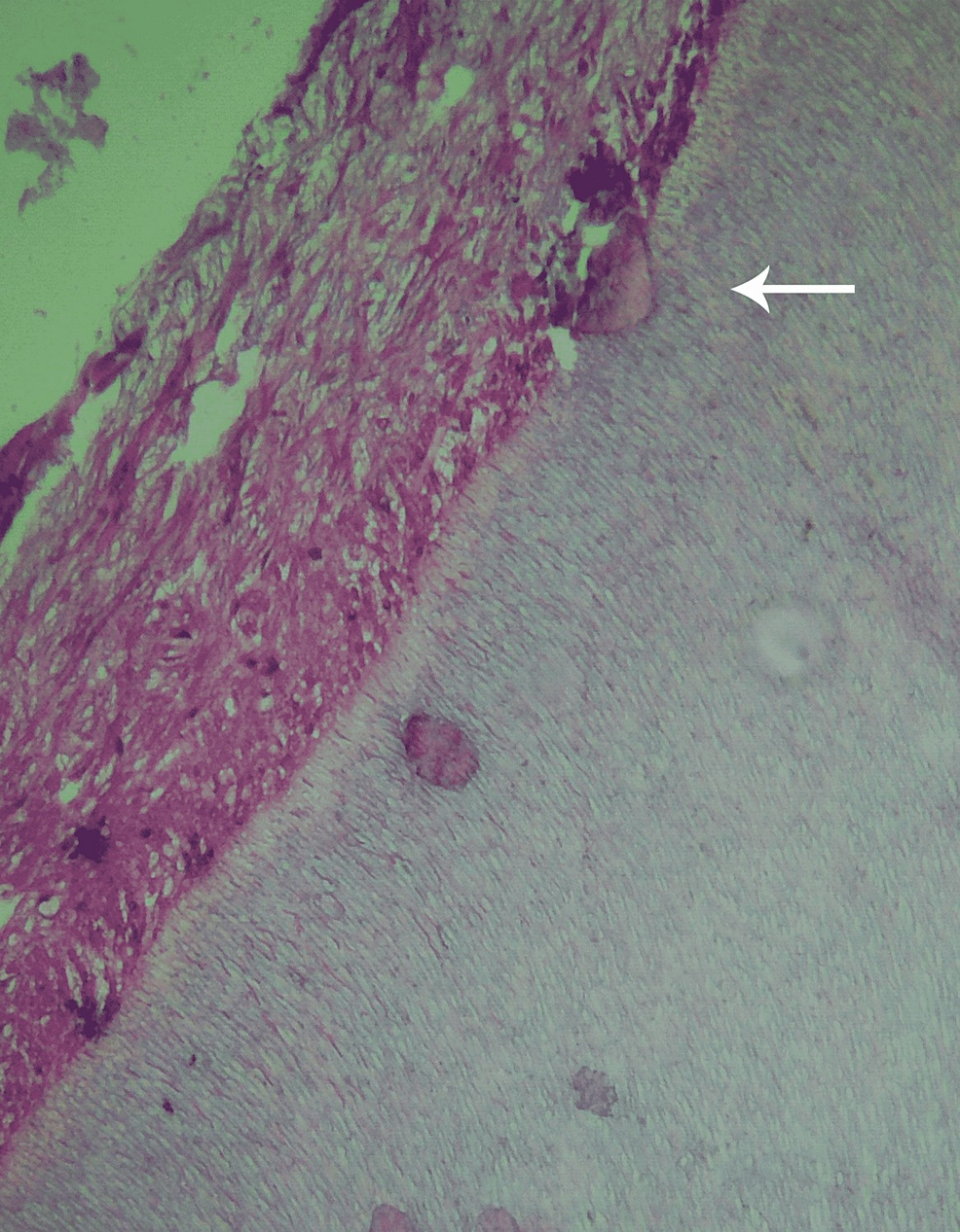
A histological preparation of a second premolar where TheraCal LC was applied showing the dentin bridge thickness (H&E stain, 100×).

The dentin bridge thickness was also examined in both groups. The TheraCal group showed a thickness greater than 0.25mm in 60% of the samples, while the other 40% had a thickness ranging from 0.1mm to 0.25 mm. The examination of the dentin bridge thickness in the MTA group showed that 70% of the samples had a thickness greater than 0.25 mm, while the remaining 30% had a thickness ranging from 0.1 mm to 0.25 mm. The minimal differences between the two thicknesses were statistically insignificant (P=0.739) (Figure 4).

Degree of pulpal inflammation

**Figure 5:**
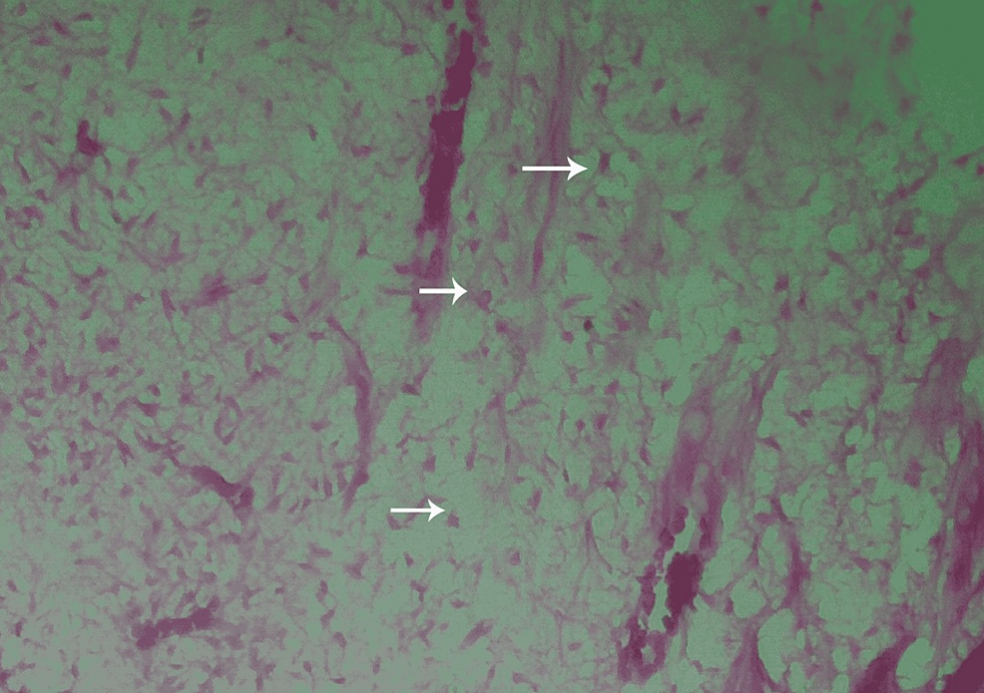
A histological preparation of a second premolar where TheraCal LC was applied showing first-degree pulpal inflammation (H&E stain, 100×).

The degree of inflammation in the TheraCal LC group was mild to moderate in 40% of the teeth and without pulpal inflammation in 60% of the teeth. The MTA group showed 10% of the patients having a degree of inflammation that was mild to moderate, while 90% of the samples showed no pulpal inflammation. This was statistically insignificant (P=0.280) (Figure 5).

Odontoblast layer

**Figure 6:**
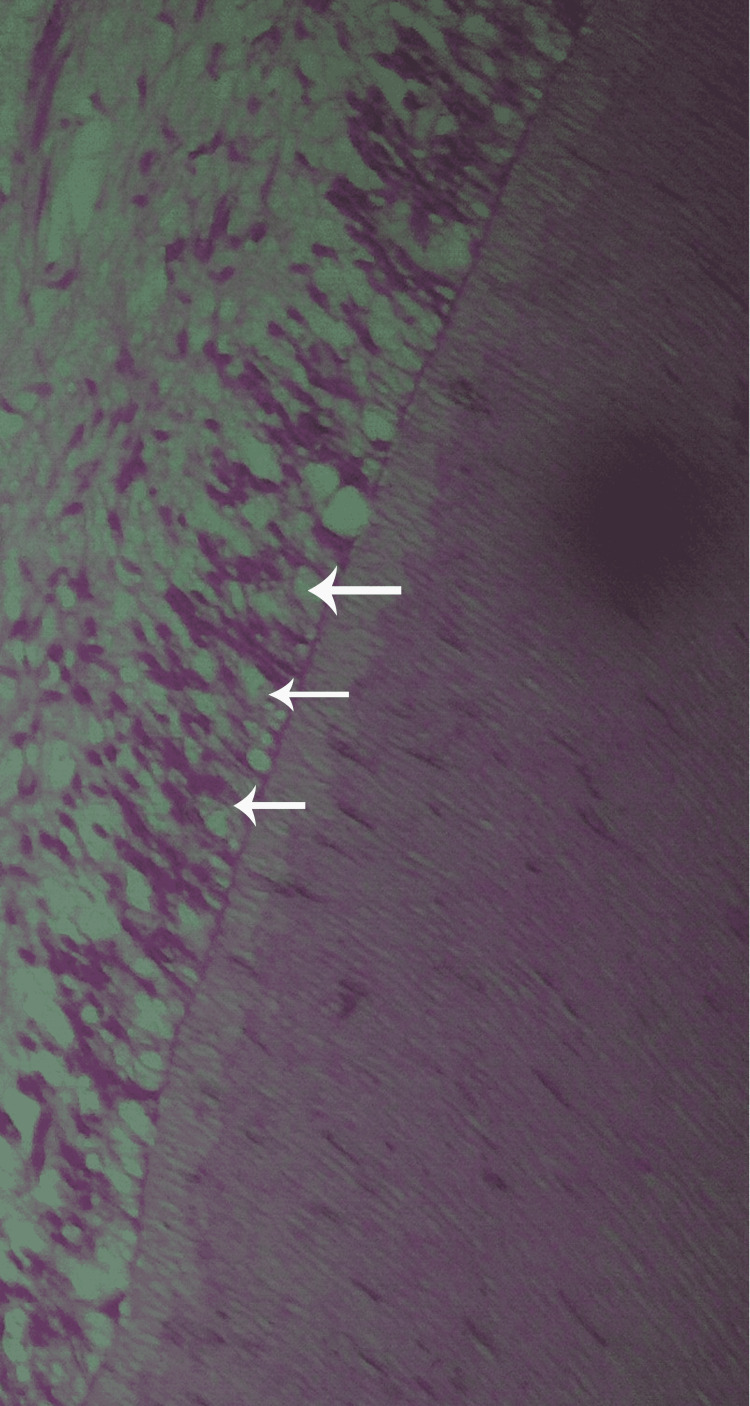
A histological preparation of a second premolar where TheraCal LC was applied showing the odonotblasts alignment (H&E stain, 100×).

The odontoblasts in the TheraCal LC group were lined up regularly in 60% of the teeth, while 40% of them showed irregularly aligned odontoblasts. In the MTA group, the odontoblasts were regularly lined up in 80%, and irregularly aligned in 20%. These differences were statistically insignificant between the groups (P=0.481) (Table 1, Figure 6).

**Table 1:** The results of using Theracal LC and MTA on direct pulp capping (DPC).

Material type	Number of teeth	Study duration	The formed dentin bridge composition	The formed dentin bridge thickness	Pulpal inflammation degree	Odonotblast layer
Theracal LC	10 Premolars	10 Weeks	80% effective tubules	<0.25 mm in 60%	No pulpal inflammation in 60%	Regular alignment in 60%
			20% defective tubules	0.1-0.25 mm in 40%	Mild to moderate inflammation in 40%	Irregular alignment in 40%
MTA	10 premolars	10 Weeks	90% effective tubules	<0.25 mm in 70%	No pulpal inflammation in 90%	Regular alignment in 80%
			10% defective tubules	0.1-0.25 mm in 30%	Mild to moderate inflammation in 10%	Irregular alignment in 20%

## Discussion

The dentin bridge was formed in all samples using MTA, which reacts with calcium oxide, water, and other fluids forming calcium hydroxide. The result (calcium hydroxide) has a high alkaline ability that is can induce odontoblasts in the dental pulp complex. This forms well-defined dentin that assists with the formation of a dentin bridge, which will eventually close the exposure [12]. This study did coincide with Faraco Junior studies in 2001, which showed a total recovery of the exposure with a tubular dentin bridge formation without any inflammation in any of the dogs’ teeth capped with MTA [5]. It also coincided with the study of Soraes in the year 1996, in which he noticed the formation of a dentin bridge in 96.43% of the samples after pulpotomy was completed using MTA [13]. Aboud and Alnehlawy, in their 2019 study, found a 93.8% success rate on the teeth that were capped using MTA [14]. Furthermore, Alarashi found in a 2007 study the histologically well-defined dentin bridge in an MTA sample after two months of placement [15].

For the dentin bridge formation, our study found that in 90% of the samples, the dentin bridge had well-defined tubules and an increase in the alkaline phosphatase, which plays a role in the precipitation of the calcified compounds, and by that, the proliferation of odontoblasts when it reaches the pulp [16]. That coincides with the results of Faraco Junior in 2001 [5], and Asgary in 2008 [17].

Most of the samples showed an absence of pulpal inflammation, this is most likely due to the biologically active properties of the MTA. It does not interfere with the pulp tissue but stays in contact on the exposure surface [18]. These results coincide with the results of Pitt Ford, who studied the reaction of pulp tissue to the MTA and calcium hydroxide when used as capping materials. It showed results of dentin bridge formation in all of the capped pulps with MTA with the absence of any inflammation [19]. The odontoblast's presence was observed in all the samples, with the majority of them found to be well aligned. This is the result of the alkaline PH formed by MTA that can induce odontoblasts [20].

All samples of the TheraCal group formed a dentin bridge. The alkaline resin-modified calcium silicate allowed the pH to rise to 10-11, which aids in pulp healing. The elevated pH will resume to its normal range after a few weeks [7,8]. This study results coincided with the study results of Kim and his colleagues in 2020. This study showed that TheraCal LC is biologically active and able to achieve an alkaline media to induce the dentin bridge [21]. Furthermore, it coincides with the results of Kunert and his colleagues in 2020, in which he noticed the formation of a dentin bridge in the TheraCal group [22].

For the dentin bridge shape, it appeared as well-defined tubules in 80% of the samples. It was found that calcium silicate releases a higher number of calcium ions when solidified, which allows the pulp cells to proliferate and increases the probability of tissue formation [16]. This coincides with our study results which showed the thickness efficiency of calcified dentin bridge in the TheraCal LC group [23].

The thickness of the formed dentin bridge was greater than 0.25 mm in more than half of the teeth, and this can be explained by the alkaline environment conserved from the TheraCal placement, which in turn aids in the formation of the dentin bridge for a period of time [8]. This supports the study of Kamal and his colleagues where an approximately 0.2 mm was hardened calcified tissue in the TheraCal group [24].

Odontoblasts were present in all the TheraCal samples, where most of them had a siege shape, thus, it was noted that TheraCal has the ability to induce odontoblasts formation like MTA [23]. This supports the results of Kamal's study, in which he found a layer of odontoblasts like cells in the newly formed calcified tissues [24].

As for pulpal inflammation, no pulpal abscesses were found. There was, however mild to moderate pulpal inflammation in half of the cases. No inflammation was present in the second half because the alkaline media prevents the proliferation of bacteria, limiting the presence of inflammation. This aligns with the study of Kamal, in which he found that pulpal inflammation was minimal in the TheraCal group [24].

## Conclusions

This study concluded that the use of both TheraCal and MTA yields a favorable and viable treatment option. The differences in the dentin bridge composition and the dentin bridge thickness produced by both treatment options yield statistically insignificant results, rendering them both similar in their effectiveness in treating pulp exposures through pulp capping. The limitations of this study were the short follow-up period which was ten weeks and studying only pathological changes on pulp tissues. Therefore, this research would be a good start to implementing other clinical studies on the same materials and observing the outcomes when they are applied for longer periods.
